# Practices and perceptions of antibiotic use in canine breeding: a survey among breeders and veterinarians

**DOI:** 10.3389/fvets.2026.1879574

**Published:** 2026-07-17

**Authors:** Lotte Spanoghe, Anouck Quaaden, Joke Lannoo, Penelope Banchi, Guillaume Domain, Florin Petrisor Posastiuc, Geert Opsomer, Ann Van Soom

**Affiliations:** 1 Department of Internal Medicine, Reproduction and Population Medicine, Faculty of Veterinary Medicine, Ghent University, Merelbeke, Belgium; 2 Department of Animal Breeding & Genomics, Wageningen University & Research, Wageningen, Netherlands

**Keywords:** antimicrobial resistance, antimicrobial use, canine reproduction, dog breeding, survey, vaginal bacterial culture

## Abstract

Antibiotic use in canine breeding, particularly prior to mating, is frequently discussed in practice despite increasing awareness of antimicrobial stewardship and the recognition that the canine vaginal tract naturally harbors a microbiome. Diagnostic uncertainty, especially regarding bacterial culture findings and *Mycoplasma* spp. detection, may contribute to antimicrobial use in this setting. This study aimed to investigate antibiotic use, bacteriological testing practices, and underlying beliefs among dog breeders and veterinarians involved in canine reproduction. Two anonymous online questionnaires targeting breeders and veterinarians were distributed in Belgium and the Netherlands between October 2025 and February 2026. The surveys collected data on antibiotic use, diagnostic practices, and perceptions regarding vaginal bacteriology and the detection of *Mycoplasma* spp. Descriptive statistics and logistic regression analyses were performed to identify factors associated with antibiotic use. A total of 426 breeders and 94 veterinarians participated. Thirty-two percent of breeders reported ever administering antibiotics around the time of breeding, most commonly prior to mating. Forty-nine percent of breeders had ever had vaginal bacteriology performed in their breeding bitches, which was strongly associated with consequent antibiotic use (OR = 4.93, 95% CI 3.14–7.90, *p* < 0.001). In contrast, most veterinarians reported never prescribing antibiotics to clinically healthy bitches, whereas 27% had done so occasionally. Breeder request, duration of professional experience and use of genital bacteriology were positively associated with antibiotic prescribing in univariable analyses. Lack of knowledge regarding the clinical relevance of *Mycoplasma* spp. was observed among both breeders and veterinarians, despite frequent testing for it and a tendency toward antibiotic treatment following detection. Antibiotic use in canine breeding is influenced by bacteriological testing, diagnostic uncertainty, and precautionary decision-making. Discrepancies between breeder-reported practices and veterinarian prescribing behavior highlight the complexity of antimicrobial use in this field. Targeted education and improved communication are essential to promote evidence-based reproductive management and responsible antimicrobial use.

## 1 Introduction

Although overall fertility in dogs is high, failure to conceive does occur and remains an important concern for both breeders and veterinarians. Within the dog breeding community, the presence of bacteria in the vaginal tract is often perceived as a major cause of failed conception, early embryonic loss, or reduced litter size (1, 2). Consequently, the administration of antibiotics prior to mating, frequently with the intention of “cleaning” the vagina, is commonly practiced (1, 2).

However, the canine vaginal tract is not sterile (3–7). Conventional culture-based methods detect bacteria in almost all healthy bitches (3, 8), but capture only a small fraction of the vaginal microbiota (9). Moreover, bacterial presence does not necessarily indicate infection (10–12). Studies have shown that species commonly identified by culture, such as *Escherichia coli, Streptococcus* spp., and *Staphylococcus* spp., are part of the normal vaginal flora in the bitch (3). In addition, culture-based studies have not consistently found differences in bacterial growth between healthy bitches and those with vaginitis, sub-, or infertility (10–12). These findings suggest that the presence of bacteria detected by routine culture is not inherently associated with disease, and routine bacteriological screening or prophylactic antimicrobial treatment is not justified in the absence of clinical signs.

Despite this, veterinarians are sometimes consulted by dog breeders regarding vaginal bacteriology and antibiotic use prior to mating (2). In clinical practice, requests for antimicrobial treatment are not uncommon, even when no clinical abnormalities are present and microbiological confirmation is lacking. Veterinarians may experience pressure to comply with such requests, particularly when breeders fear financial or emotional losses associated with unsuccessful breeding attempts. This situation is further complicated by the evolving understanding of the canine reproductive microbiome (9, 13–17), a field that has only recently gained attention.

The use of antibiotics without clear indication raises multiple concerns. From an individual patient perspective, antimicrobial exposure can cause adverse effects, including toxicity and hypersensitivity reactions, thereby affecting animal welfare. In addition, inappropriate antibiotic use may disrupt the vaginal microbiota and impair fertility (18–20). These dysbiotic effects may extend to other microbial niches, including the endometrium (9) and gut (21, 22), and may also affect early-life microbial colonization in puppies, as their pioneer microbiota partly derives from the dam's vaginal microbiota (23).

Beyond these direct effects on animal health, antimicrobial overuse in companion animals contributes to antimicrobial resistance (24, 25). From a One Health perspective, dogs may serve as reservoirs for resistant bacteria and resistance genes with potential transmission to humans and the environment (26–28). Antimicrobial stewardship is therefore essential in small animal reproduction, particularly in breeding settings where routine antimicrobial exposure may occur.

Especially in canine reproduction, *Mycoplasma* spp. occupy a particularly controversial position (15, 29–31). These organisms are frequently detected in the genital tract of both bitches (8, 9, 14, 15, 32) and stud dogs (29, 33), yet their clinical relevance remains uncertain (15, 29, 30). Positive *Mycoplasma* spp. test results are often interpreted as pathological findings, leading to treatment, breeding restrictions, or exclusion of animals from breeding programs, even in the absence of clinical signs (6, 15). The lack of consensus regarding the interpretation of bacteriological and *Mycoplasma* spp. findings contributes to variability in clinical decision-making and may further drive unnecessary antimicrobial use (6, 15).

Although concerns regarding antimicrobial use in canine breeding have been raised previously, objective data on current practices remain limited. Little is known about how commonly breeders administer antibiotics prior to mating, which motivations underlie these practices, and how veterinarians respond to breeder requests. Furthermore, discrepancies may exist between breeder-reported practices and veterinarian-reported prescribing behavior, suggesting potential communication gaps.

The present study aims to address these knowledge gaps by investigating antibiotic use in canine breeding from both breeder and veterinarian perspectives. Using two structured questionnaires, this cross-sectional study evaluates the prevalence of (prophylactic) antibiotic use in breeding bitches, the beliefs surrounding vaginal bacteria including *Mycoplasma* spp., and the diagnostic and prescribing practices of veterinarians. By placing these findings within the current understanding of the canine vaginal microbiome, this study aims to support evidence-based reproductive management and responsible antimicrobial use in canine breeding.

## 2 Materials and methods

Data were collected using two anonymous online questionnaires between October 2025 and February 2026. One questionnaire targeted veterinarians involved in canine reproductive care, and the second targeted dog breeders. Both surveys were developed using Qualtrics (Qualtrics, Provo, UT, USA) and made available through Ghent University. Participation was voluntary, and responses were stored anonymously after respondents proceeded beyond the introductory information page, which outlined the purpose of the study and data protection measures.

The veterinarian questionnaire targeted practitioners who regularly or occasionally manage breeding dogs, including breeding management, pregnancy monitoring, parturition assistance, and post-partum care. The breeder questionnaire targeted professional breeders as well as private dog owners with occasional litters. The surveys were initially developed in Dutch and subsequently translated into French and English. Conditional logic was applied so that selected questions were displayed only when relevant based on previous answers.

Participants were recruited using multiple dissemination strategies to ensure broad professional and geographic coverage within Belgium and the Netherlands. Survey links were distributed via email through the Dutch-speaking Regional Council of the Belgian Veterinary Council (Nederlandstalige Gewestelijke Raad van de Orde der Dierenartsen, NGROD), veterinary practices in the Netherlands, and breeder associations, and were additionally shared via social media posts from the Clinic of Small Animal Reproduction (Ghent University) and private accounts. QR codes providing direct access to the questionnaires were also presented at relevant professional and educational meetings, including a breeder information evening at the Clinic of Small Animal Reproduction and at Expovet, a local fair for veterinarians and students in veterinary medicine.

The veterinarian questionnaire addressed professional background, antibiotic use in canine reproduction, diagnostic approaches to vaginitis and fertility disorders, interactions with breeders, and perceptions and management of *Mycoplasma* spp. Questions focused on the frequency and timing of antibiotic use throughout the reproductive cycle, including preventive use in clinically healthy bitches, diagnostic strategies for suspected infections, and antibiotic selection in cases of vaginitis, vaginal discharge, and (sub)fertility problems. Additional items explored breeder-requested antibiotic use and veterinarians' interpretation of bacteriological findings, including *Mycoplasma* spp. detection in bitches and stud dogs.

The breeder questionnaire collected background information on breeding activity, including experience, breeds, number of litters per year, and mating methods. Further questions focused on antibiotic use in breeding bitches, including indications, timing, treatment duration, and source of antibiotics, as well as whether similar strategies were applied across multiple animals and whether perceived differences in reproductive outcomes were observed. The final part addressed bacteriological testing practices and knowledge and perceptions of *Mycoplasma* spp. in relation to fertility and breeding decisions.

Both questionnaires consisted of single-response, multiple-response, and open-ended questions, with mandatory responses for all items. An optional free-text field at the end of each questionnaire allowed respondents to provide additional comments. The complete questionnaires are provided as Supplementary Appendices 1(breeders) and 2 (veterinarians).

The survey utilized a forced-response format, requiring participants to answer each item before proceeding. However, respondents could discontinue the questionnaire at any time; in such cases, responses provided up to the point of discontinuation were recorded, resulting in incomplete questionnaires with missing data primarily in the final sections. Descriptive analyses were performed on an item-by-item basis. For inferential analyses, only respondents with complete data for the variables included in each model were analyzed.

Data were exported from Qualtrics to Microsoft Excel^®^ (Microsoft Corporation, Redmond, WA, USA). Descriptive analyses were performed using absolute and relative frequencies.

Statistical analyses and graphical representations were conducted R version 4.3.1 (R Foundation for Statistical Computing, Vienna, Austria). Associations between categorical variables were evaluated using Pearson's chi-square tests. When appropriate, uni- and multivariable logistic regression analysis was performed to assess predictors of antibiotic use and vaginal bacteriological testing. Statistical significance was set at *p* < 0.05.

## 3 Results

### 3.1 Breeder responses

#### 3.1.1 Respondent characteristics

**Figure 1:**
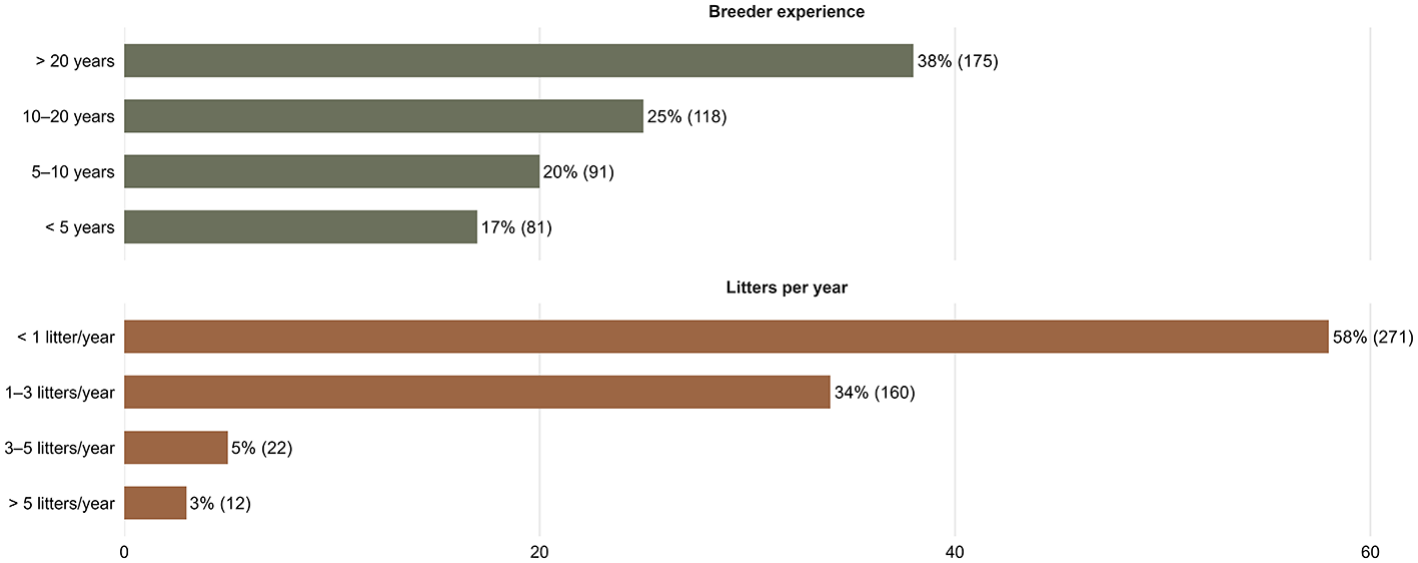
Breeding experience of respondents and number of litters per year in percentage and absolute numbers.

A total of 533 responses were received, 426 fully completed. Most respondents were Dutch breeders (76%; *n* = 354/465) and 24% Belgian (*n* = 111/465), representing 175 breeds; Dachshunds, Poodles, and Golden Retrievers were most common. Breeding experience and litters per year are shown in Figure 1.

#### 3.1.2 Use of genital bacteriology

**Figure 2:**
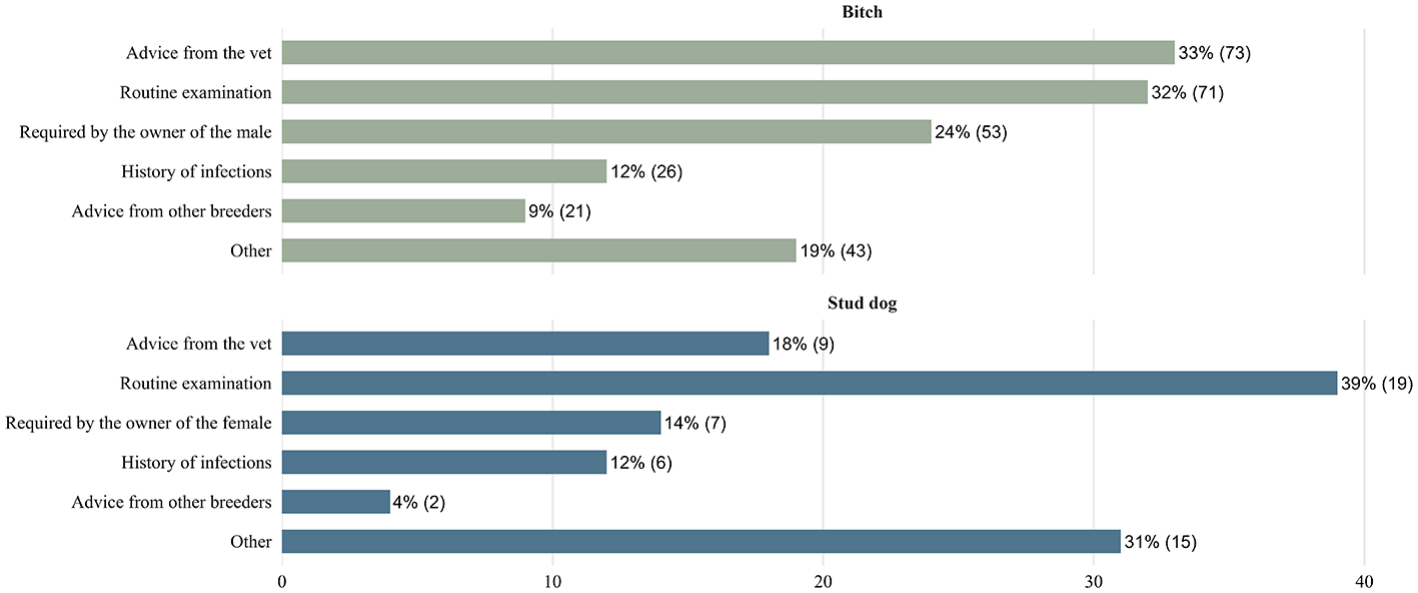
Reasons for performing genital bacteriological testing in bitches and stud dogs, presented as percentages and absolute numbers.

Vaginal bacteriology was performed by 49% of breeders (*n* = 222/449), while bacteriological testing of males was mentioned by 11% of the respondents (*n* = 49/449). The reported reasons for performing bacterial testing in both bitches and males are summarized in Figure 2. Breeders main reasons for selecting “other” were infertility in both sexes and in the case of stud dogs export of semen.

**Figure 3:**
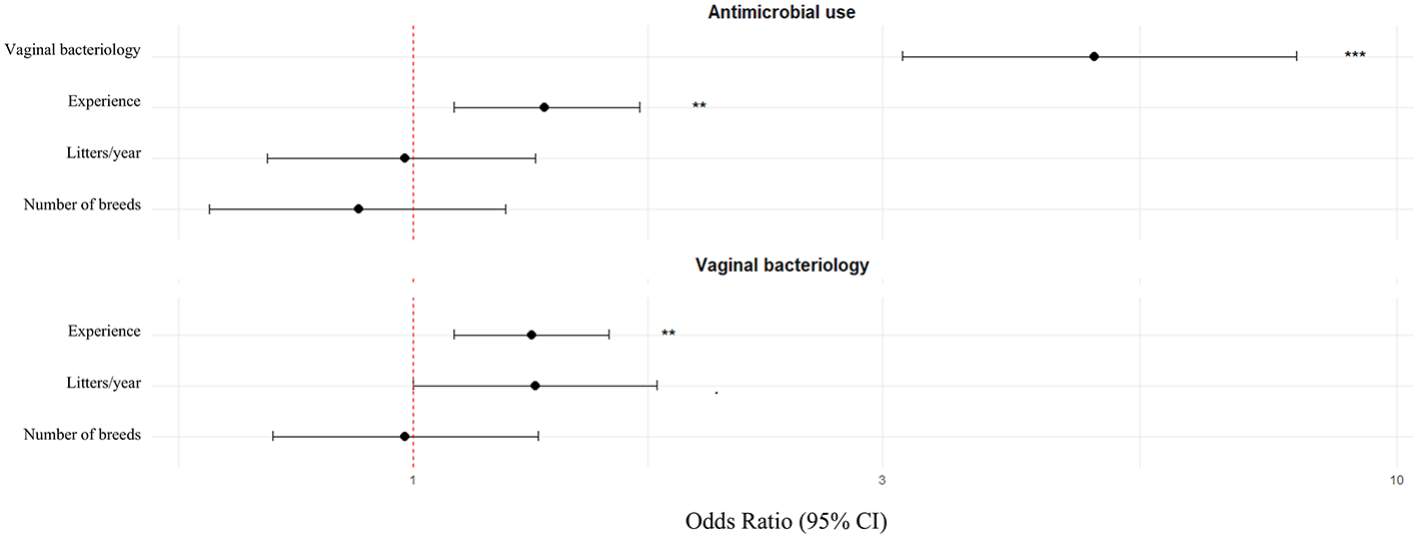
Odds ratios (ORs) and 95% confidence intervals from multivariable logistic regression models for factors associated with antimicrobial use **(top)** and performance of vaginal bacteriology **(bottom)** by breeders. The dashed line indicates OR = 1. Vaginal bacteriology and breeder experience were significantly associated with antimicrobial use, while experience was also associated with performing bacteriology. Statistical significance is indicated as *p < 0.05, **p < 0.01, ***p < 0.001; · indicates a trend (·*p* < 0.10).

In univariable analyses, experience and breeder size (number of litters per year) were positively associated with the use of vaginal bacteriology, whereas number of breeds managed was not. In multivariable analysis, only experience remained significantly associated [OR 1.32, 95% CI (1.10–1.58), *p* = 0.003], with a borderline effect of number of litters per year [OR 1.33, 95% CI (1.00–1.77), *p* = 0.051] (Figure 3).

#### 3.1.3 Antibiotic use in breeding

**Figure 4:**
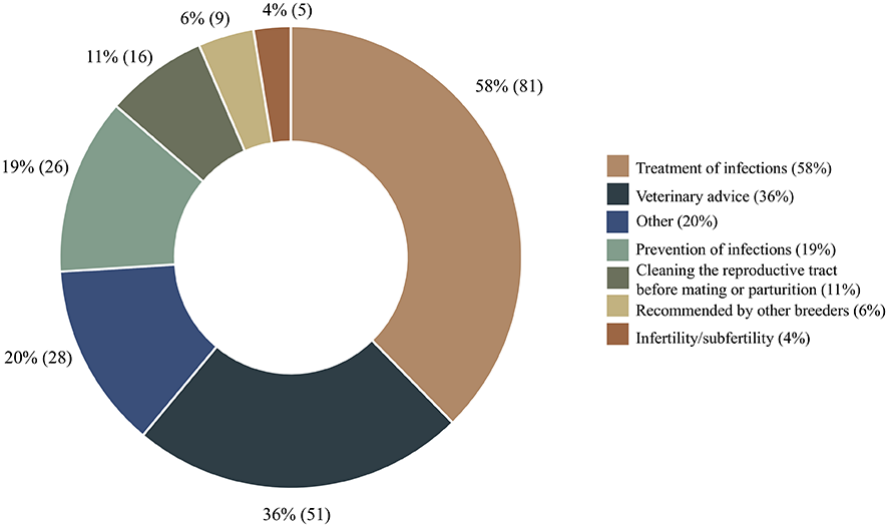
Indications for antibiotic use among breeders who administered antibiotics in dog breeding.

Overall, 32% of breeders (*n* = 148/460) indicated that they had ever administered antibiotics to their bitch in relation to breeding, mainly before mating (63%; *n* = 88/140) and during lactation (27%; *n* = 38/140). Reasons why antibiotics were used are reported in Figure 4. Amoxicillin and amoxicillin-clavulanic acid were the most used; 32% did not know which antibiotic had been used (*n* = 45/140). Most respondents reported short treatment durations, with 55% administering antibiotics for less than 7 days (*n* = 77/140) and 36% for 7–14 days (*n* = 50/140), while longer courses of 15–21 days (7%; *n* = 10/140) or more than 21 days (4%; *n* = 5/140) were less commonly used.

Breeders who had vaginal bacteriology performed on their bitches were substantially more likely to administer antibiotics than those who did not. In multivariable logistic regression, having bacteriology performed remained strongly associated with antibiotic use, with almost five-fold higher odds of antibiotic administration among breeders whose bitches underwent bacteriology compared with those who did not [OR = 4.93, 95% CI (3.14–7.90), *p* < 0.001].

Increasing breeder experience was also associated with higher odds of antibiotic use [OR = 1.36 per category increase, 95% CI (1.10–1.70), *p* = 0.005], indicating a progressive increase in antibiotic administration with greater experience. In contrast, number of litters per year and number of breeds managed were not significantly associated with antibiotic use in the adjusted model (Figure 3).

#### 3.1.4 Knowledge and perceptions of Mycoplasma spp.

Sixty-two percent of responding breeders were familiar with *Mycoplasma* infections (*n* = 275/447), but nearly half were unsure of their impact on fertility (*n* = 126/262). *Mycoplasma* spp. PCR testing was reported by 34% of breeders in bitches (*n* = 88/262) and 16% in stud dogs (*n* = 31/188). About half indicated they would administer antibiotics following *Mycoplasma* spp. detection [bitches 52% (*n* = 136/262), stud dogs 46% (*n* = 120/262)].

### 3.2 Veterinary responses

#### 3.2.1 Respondent characteristics

**Figure 5:**
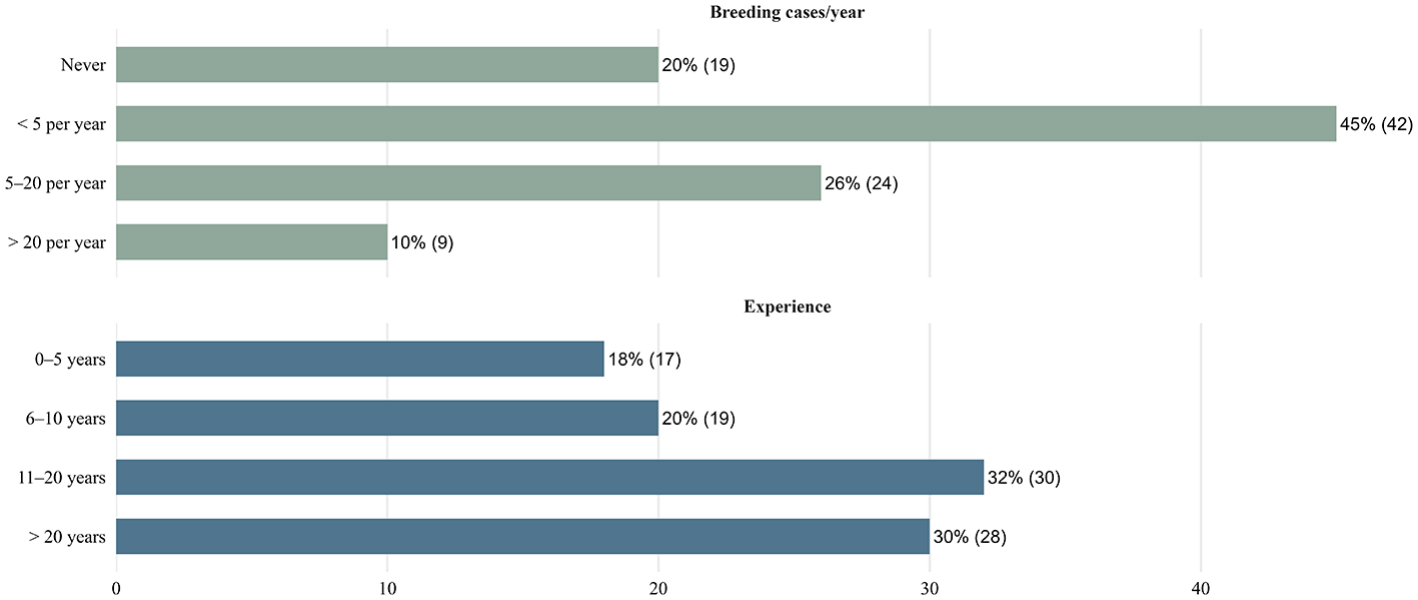
Distribution of respondents by clinical experience and annual frequency of breeding case management (*n* = 94). Percentages and absolute numbers are presented. Practitioners who indicated that they never see breeding cases were excluded from further analysis.

A total of 131 veterinarians accessed the survey; 65 completed it. Among 94 providing demographics, 54% practiced in Belgium (*n* = 51/94) and 46% in the Netherlands (*n* = 44/94). Professional experience and breeding caseload are presented in Figure 5. Practitioners indicating that they never see breeding cases were excluded from further analysis (*n* = 19).

#### 3.2.2 Antibiotic use in breeding

**Figure 6:**
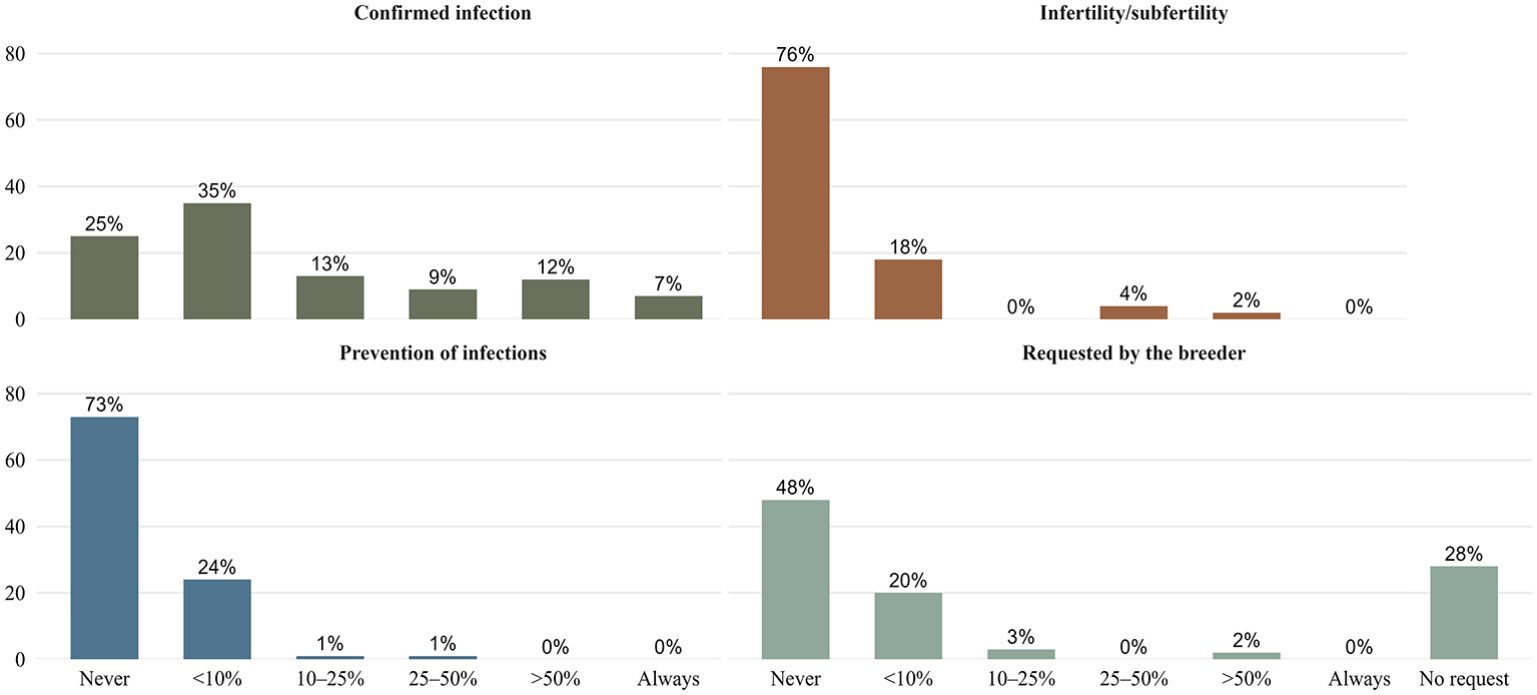
Reported proportion of breeding cases in which veterinarians prescribe antibiotics for specific indications (prevention of infection, confirmed infection, infertility/subfertility, or at the breeder's request). In the “breeder's request” panel, “no request” indicates the proportion of veterinarians who reported never having received a request from breeders to prescribe antibiotics.

Twenty-seven percent of veterinarians (*n* = 20/73) reported prescribing antibiotics to healthy breeding bitches, mostly prior to mating (63%; *n* = 12/19) and around parturition (32%; *n* = 6/19). Amoxicillin-clavulanic acid was most prescribed (74%; *n* = 14/19). Figure 6 presents the reported frequency with which veterinarians prescribe antibiotics for specific breeding-related indications.

#### 3.2.3 Requests from breeders

Requests from breeders regarding vaginal bacteriology and preventive antibiotic use in healthy breeding bitches were generally infrequent (Table 1).

**Table 1:** Veterinarian's reported frequency of breeder requests for vaginal bacteriology and preventive antibiotic use in healthy breeding bitches.

Request type	Never	< 10%	10–25%	25–50%	>50%
Vaginal bacteriology	53%	32%	8%	2%	5%
Advice on antibiotic use around mating/parturition	27%	58%	10%	5%	–
Preventive use of antibiotics (no clear indication)	48%	35%	17%	–	–

Response categories indicate the proportion of breeders making such requests (Never, < 10%, 10–25%, 25–50%, >50%); values are percentages of responding veterinarians.

#### 3.2.4 Mycoplasma management

Fifty-nine percent of responding veterinarians were unsure whether vaginal *Mycoplasma* spp. causes infertility (*n* = 41/70). When *Mycoplasma* spp.-positive animals were presented, 40% of respondents reported they would administer antibiotics in male dogs (*n* = 22/55) and 48% in female dogs (*n* = 27/56). Among the veterinarians who were uncertain about the role of *Mycoplasma* spp. in infertility, 43.9% still reported that they would administer antibiotics in *Mycoplasma*-positive animals (*n* = 18/41).

#### 3.2.5 Factors associated with antibiotic prescription

**Figure 7:**
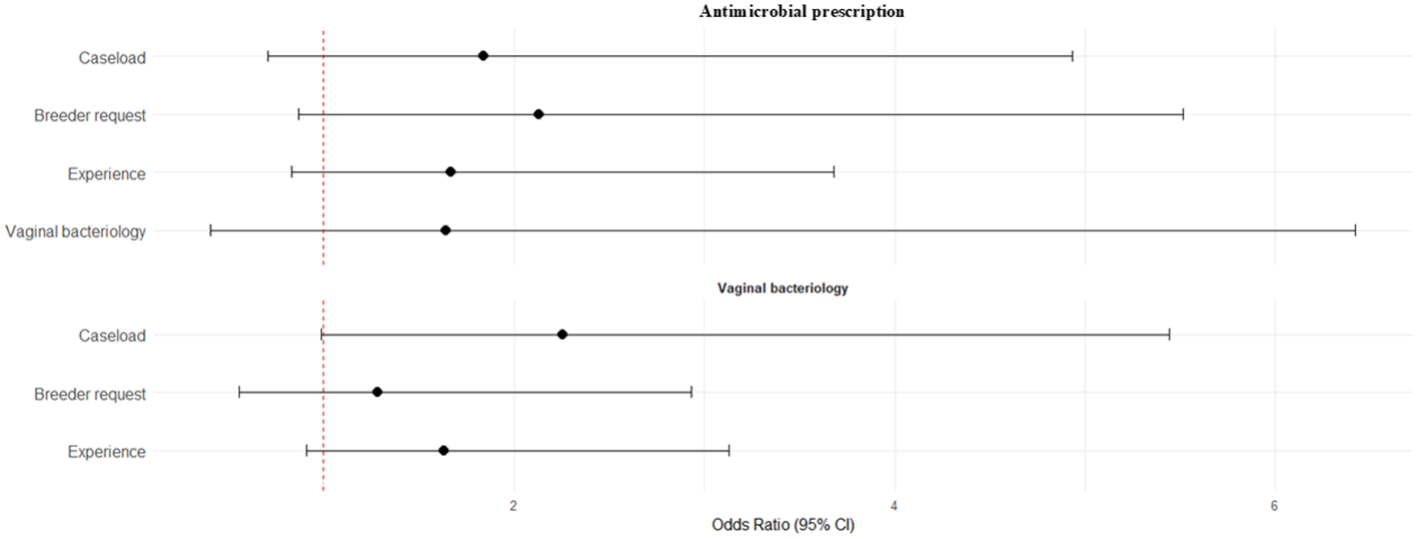
Odds ratios (ORs) and 95% confidence intervals from multivariable logistic regression models for factors associated with antimicrobial prescription (top) and performance of vaginal bacteriology (bottom) among veterinarians. The dashed line indicates OR = 1. Predictor variables include experience, caseload, breeder request frequency and vaginal bacteriology use. In the antimicrobial prescription model, all predictors showed positive but non-significant associations. In the vaginal bacteriology model, caseload showed a trend toward association with bacteriology use (*p* < 0.10), while experience and breeder request were not significantly associated.

Breeder request frequency [OR 2.5, 95% CI (1.14–5.89), *p* = 0.027] and experience [OR 1.86, 95% CI (1.02–3.36), *p* = 0.042] were positively associated with antibiotic prescription. Performing genital bacteriology was also associated with higher antibiotic prescription (*p* = 0.0487). In multivariable logistic regression, all tested variables showed positive but non-significant associations with antibiotic prescription (Figure 7).

## 4 Discussion

This study investigated antibiotic use and bacteriological testing practices in canine breeding from both breeder and veterinarian perspectives. Antibiotic administration in breeding bitches was relatively common, with approximately one-third of breeders reporting use. Bacteriological testing was also widely performed, with nearly half of breeders reporting vaginal bacteriology, often as routine screening rather than in response to clinical signs. Among both breeders and veterinarians, antibiotic administration was positively associated with the use of vaginal bacteriology. In contrast, most veterinarians reported that they have never prescribed antibiotics to a healthy breeding bitch, although a minority (27%) reported having done so. In addition, considerable uncertainty was observed regarding the clinical relevance of *Mycoplasma* spp. among both groups.

A key finding of this study is the strong association between vaginal bacteriological testing and antibiotic administration among breeders. Breeders whose bitches underwent bacteriological testing were about five times as likely to administer antibiotics. This suggests that concerns about bacterial presence or beliefs in the benefits of prophylactic treatment may drive both testing and treatment. These findings align with previous reports indicating that detection of bacteria in the canine reproductive tract often leads to antimicrobial use, despite the fact that bacterial presence does not necessarily indicate infection (2, 3, 6). Given the diverse vaginal microbiota in healthy bitches (9, 13–17) and the frequent detection of bacteria in clinically normal animals (3–7), bacteriological findings may be misinterpreted as pathological. Importantly, this pattern was also observed among veterinarians, indicating that the presence of bacteria alone may be incorrectly equated with infection, prompting antimicrobial treatment in the absence of clinical signs. This finding is of particular concern, as it suggests a gap between current knowledge of the normal vaginal microbiome and clinical decision-making. Similar patterns have been reported in Germany, where microbiological testing is common and positive cultures frequently result in treatment or requests for treatment from breeders (2). Notably, 13.8% of veterinarians in that study indicated that they would administer antibiotics based solely on a positive culture result (2).

Antibiotic use in breeding bitches was reported by approximately 32% of breeders in the present study, often in relation to mating. A survey conducted in the United States reported comparable findings, with 23.5% of breeders indicating routine antibiotic use during pregnancy (1). Although differences in study design limit direct comparison, both studies suggest that non-therapeutic, precautionary antimicrobial use occurs in a subset of breeding programs.

In the present study, antibiotics were most frequently administered prior to mating. Reported indications included treatment of infections, prevention of infections, and “cleaning” of the vagina. While treatment of infection was the most common reason, this category may include cases where treatment was initiated following positive bacteriological testing rather than clear clinical signs. Notably, the explicit belief that antibiotics can be used to “clean” the reproductive tract of otherwise healthy animals highlights an important misconception regarding the role of the vaginal microbiota and the indications for antimicrobial use. This supports the idea that diagnostic testing and interpretation of results play an important role in driving antimicrobial use.

Veterinarians in this study reported lower levels of antibiotic use than suggested by breeder responses. Most veterinarians (73%) indicated that they never prescribe antibiotics to clinically healthy dogs. This contrasts with findings from Germany, where a smaller proportion of veterinarians (38.1%) reported avoiding antimicrobial use in the absence of clinical signs (2). These differences may reflect variation in prescribing practices between countries or differences in study populations. In Belgium, stricter regulations introduced in September 2024 may also contribute to more cautious prescribing. These regulations require bacteriological testing before the use of critically important antibiotics and prohibit any prophylactic antibiotic use (34, 35), potentially reinforcing antimicrobial stewardship. Similarly, in the Netherlands, antimicrobial use in companion animals is regulated under strict national legislation, including requirements for veterinary prescription, clinical justification, and controlled use of antimicrobials, which support prudent prescribing practices (36).

A slight difference between breeder-reported antibiotic use and veterinarian-reported prescribing was observed; however, direct comparison between these groups is inherently limited, as they do not represent matched populations. Therefore, discrepancies are expected and should be interpreted with caution. Social desirability bias may have contributed, particularly among veterinarians, potentially leading to underreporting of prescribing practices that deviate from current guidelines. In addition, a relatively high drop-out rate among veterinarians who accessed the survey may have influenced the final sample, potentially reflecting reluctance to report practices that could be perceived as non-compliant with antimicrobial stewardship recommendations. The relatively small number of veterinarian respondents may further limit representativeness. Study populations also differed, as most breeders were Dutch, whereas veterinarians were equally Belgian and Dutch.

In the present study, breeder requests for antibiotics were positively associated with prescribing behavior in univariable analysis, although this association was no longer significant after adjustment for other practice-related variables. Nevertheless, breeder influence on treatment decisions has been reported previously. In the previously cited survey among veterinarians in Germany, more than 50% of respondents indicated that owner request was a reason for performing bacteriological examination of the bitch, and 62% reported that breeders had requested antibiotic treatment for their bitch, most commonly for prophylactic use or because it was considered usual practice (2). Similarly, a qualitative study among Dutch companion animal veterinarians highlighted that breeders can be particularly demanding with regard to antimicrobial prescribing, with some veterinarians reporting that breeders believe they know when antibiotics are needed (37).

Misalignment between veterinarian and client perceptions may also play a role. Veterinarians may perceive pressure to prescribe antibiotics even when clients do not express explicit expectations, with expectations often inferred from indirect cues or assumptions about client satisfaction (38). However, evidence suggests that pet owners themselves generally report low expectations for antibiotic prescriptions and tend to defer to veterinary expertise, highlighting a potential mismatch in perceptions (38). In addition, time constraints and workload pressures have been associated with increased antibiotic prescribing, likely because prescribing is more time-efficient than pursuing alternatives such as additional diagnostics, detailed discussion, or client education (39). In reproductive management, risk aversion may further contribute to prescribing, as veterinarians aim to avoid reproductive failure and client dissatisfaction (2).

These findings are consistent with a recent systematic review examining determinants of antibiotic prescribing among veterinarians, which identified client expectations and demand as common factors influencing antimicrobial use (40). In many cases, veterinarians reported prescribing antibiotics in response to perceived client expectations or pressure, sometimes even when treatment was not clearly indicated (41, 42). Client demand is often linked to limited awareness of antimicrobial resistance and appropriate antibiotic use, highlighting the importance of communication and education in antimicrobial stewardship (43–45).

Professional experience may also influence antibiotic prescribing behavior. In the present study, greater experience was positively associated with antibiotic use among both veterinarians and breeders. Previous research reports mixed findings: some studies associate younger or less experienced veterinarians with higher antibiotic use, possibly due to lower diagnostic confidence or greater susceptibility to client pressure (37, 40, 46), whereas others report increased prescribing among more experienced practitioners (42, 44, 47–50), potentially reflecting differences in training and historically accepted practices when antimicrobial stewardship received less emphasis.

Uncertainty regarding the clinical relevance of bacteriological findings, particularly *Mycoplasma* spp., was evident in this study. *Mycoplasma* spp. are commonly detected in the canine reproductive tract and are generally considered part of the normal genital microbiota (15, 29, 30), although some species have historically been suspected to play a role in reproductive disease. Earlier studies (1970–2000) reported the isolation of *Mycoplasma* spp. in dogs with cystitis (51, 52) and suggested that genital tract disease in both males and females could be induced experimentally following infection (53, 54). In addition, a case report described the presence of *Mycoplasma* spp. in a kennel experiencing fertility problems (31), although such observation does not provide any evidence for a causal relationship. More recent studies, however, comparing fertile and infertile dogs have not identified a statistically significant difference in the prevalence of *Mycoplasma* spp. (15, 29, 30).

In line with this ambiguity, many breeders and veterinarians in the present study were familiar with *Mycoplasma* spp.; however, nearly half were unsure whether its presence affects fertility. Notably, among those who were uncertain, 44% indicated that they would still administer antibiotics following detection. This pattern suggests that antibiotic use may be driven by diagnostic uncertainty and precautionary reasoning rather than clear clinical indications. Similar behavior has been described in Belgian, British and Australian veterinarians, who may prescribe antibiotics “out of caution” in uncertain situations (39, 55, 56).

Similar patterns were observed among breeders. In the present study, both antibiotic administration and vaginal bacteriological testing increased significantly with breeding experience. This may reflect the persistence of historically established breeding practices, as informal comments provided in the questionnaire indicated that some breeders considered antibiotic administration prior to mating to be a traditional approach.

Finally, antimicrobial use in breeding animals should be considered within the broader context of antimicrobial resistance. It primarily selects for resistance within the treated animal's own microbiota, increasing the risk of subsequent antimicrobial-resistant infections. Resistant bacteria and genes may also spread between animals, humans or the environment (18–20, 57, 58).

Evidence from recent studies suggests that continuing professional education and access to updated scientific knowledge are associated with more prudent antibiotic prescribing among veterinarians (40). Targeted education should not only increase awareness of antimicrobial resistance, but also address key drivers of inappropriate use identified in this study, particularly the tendency to treat the presence of bacteria rather than clinical infection. Improving understanding of when antimicrobial therapy is indicated, among both veterinarians and breeders, may therefore play an important role in supporting evidence-based antimicrobial use in canine breeding.

As a questionnaire-based study, methodological limitations must be acknowledged. First, voluntary participation may have introduced selection bias, potentially resulting in overrepresentation of veterinarians interested in antimicrobial stewardship and conscientious breeders. Large-scale breeders may therefore be underrepresented. Second, reliance on self-reported data carries an inherent risk of recall bias and social desirability bias, whereby respondents may have underreported practices perceived as unfavorable. Finally, the cross-sectional design of this study precludes the establishment of causal relationships between the variables considered.

Despite these limitations, this study has several strengths. Notably, it includes both breeders and veterinarians, allowing for comparison of perspectives and providing a more comprehensive understanding of factors influencing antimicrobial use in canine reproduction. In addition, it addresses a relatively underexplored area and offers valuable insights into real-world practices, which may help inform targeted antimicrobial stewardship strategies in this field.

Future research should further explore the canine vaginal microbiome, with particular attention to the role of *Mycoplasma* spp. and other bacteria in vaginal health and fertility outcomes. Improved understanding of the clinical relevance of bacterial findings versus normal colonization is needed to support evidence-based decision-making. In addition, qualitative studies could help elucidate the beliefs and contextual factors underlying antimicrobial prescribing behaviors in both veterinarians and breeders.

## 5 Conclusion

This study indicates that antibiotic use in canine breeding is closely associated with vaginal bacteriological testing, despite the fact that bacterial detection does not necessarily reflect infection. Uncertainty in interpreting these findings, particularly regarding *Mycoplasma* spp., may contribute to precautionary antimicrobial use in clinically healthy bitches. These results highlight the need for improved education and antimicrobial stewardship in canine reproduction, as well as further research into the normal vaginal microbiome and its clinical relevance. Such efforts are important to support evidence-based reproductive management and help reduce unnecessary antimicrobial use.

## Data Availability

The raw data supporting the conclusions of this article will be made available by the authors, without undue reservation.

## References

[1] Simon BJ Weese JS Schick AE Lewis TP 2nd. Breeder-reported patterns of antimicrobial use and point prevalence of methicillin-resistant staphylococcus spp among breeding bitches in the southwestern United States. Can Vet J. (2020) 61:1273–7. 33299242 PMC7659873

[2] Rojahn A Leps AS Goericke-Pesch S. German veterinarians asked: a cross-sectional study on microbiological examination and antimicrobial use in canine reproductive medicine. Front Vet Sci. (2025) 12:1645496. doi: 10.3389/fvets.2025.1645496 40800233 PMC12339326

[3] Leps AS Klein B Schneider M Meyer C Šoba A Simon C . The canine vaginal flora: a large-cohort retrospective study. Vet Sci. (2024) 11:55. doi: 10.3390/vetsci11020055 38393073 PMC10892940

[4] Groppetti D Pecile A Barbero C Martino PA. Vaginal bacterial flora and cytology in proestrous bitches: role on fertility. Theriogenology. (2012) 77:1549–56. doi: 10.1016/j.theriogenology.2011.11.022 22289216

[5] Bjurström L Linde-Forsberg C. Long-term study of aerobic bacteria of the genital tract in breeding bitches. Am J Vet Res. (1992) 53:665–9. doi: 10.2460/ajvr.1992.53.05.665 1524290

[6] Banchi P Spanoghe L Maes D Morrell J Van Soom A. The reproductive microbiome in dogs: Friend or foe? Vet J. (2024) 304:106100. doi: 10.1016/j.tvjl.2024.106100 38484870

[7] Watts JR Wright PJ Whithear KC. Uterine, cervical and vaginal microflora of the normal bitch throughout the reproductive cycle. J Small Anim Pract. (1996) 37:54–60. doi: 10.1111/j.1748-5827.1996.tb01936.x 8656593

[8] Schäfer-Somi S Lechner D Tichy A Spergser J. The cultivable bacteria colonizing canine vagina during proestrus and estrus: a large-scale retrospective study of influencing factors. Animals. (2024) 14:3460. doi: 10.3390/ani14233460 39682423 PMC11640309

[9] Lyman CC Holyoak GR Meinkoth K Wieneke X Chillemi KA DeSilva U. Canine endometrial and vaginal microbiomes reveal distinct and complex ecosystems. PLoS One. (2019) 14:e0210157. doi: 10.1371/journal.pone.0210157 30615657 PMC6322750

[10] Bjurström L. Aerobic bacteria occurring in the vagina of bitches with reproductive disorders. Acta Vet Scand. (1993) 34:29–34. doi: 10.1186/BF03548220 8342462 PMC8112530

[11] Golińska E Sowińska N Tomusiak-Plebanek A Szydło M Witka N Lenarczyk J . The vaginal microflora changes in various stages of the estrous cycle of healthy female dogs and the ones with genital tract infections. BMC Vet Res. (2021) 17:8. doi: 10.1186/s12917-020-02710-y 33407480 PMC7789644

[12] Jagódka D Kaczorek-Łukowska E Graczyk R Socha P. Vaginal aerobic bacteria of healthy bitches and those with fertility problems. Pol J Vet Sci. (2023) 26:733–9. doi: 10.24425/pjvs.2023.148293 38088743

[13] Hu J Cui L Wang X Gao X Qiu S Qi H . Dynamics of vaginal microbiome in female beagles at different ages. Res Vet Sci. (2022) 149:128–35. doi: 10.1016/j.rvsc.2022.05.006 35779348

[14] Rota A Corrò M Patuzzi I Milani C Masia S Mastrorilli E . Effect of sterilization on the canine vaginal microbiota: a pilot study. BMC Vet Res. (2020) 16:455. doi: 10.1186/s12917-020-02670-3 33228646 PMC7684734

[15] Leps AS Packeiser EM Schwens C Stoelcker B Doric S Wirkner M . The canine vaginal microbiome during heat and fertility in healthy breeding dogs. PLoS One. (2025) 20:e0321683. doi: 10.1371/journal.pone.0321683 40293987 PMC12036845

[16] Spanoghe L Domain G Posastiuc F Hettiarachchi A Panattoni A Theuns S . Toward standardized methods in canine vaginal microbiome research: evaluation of storage, host DNA depletion, and database selection. Microbiol Spectr. (2025) 13:e0058325. doi: 10.1128/spectrum.00583-25 40626776 PMC12323338

[17] Gronsfeld V Brutinel F Egyptien S Porsmoguer C Hamaide A Taminiau B . Evaluation of the vaginal and urinary microbiota of healthy cycling bitches. BMC Vet Res. (2024) 20:315. doi: 10.1186/s12917-024-04104-w 39010076 PMC11247753

[18] Hiratsuka D Matsuo M Hirota Y. The reproductive tract microbiome and female fertility: dysbiosis, disease links, and emerging therapeutic strategies. Fertil Steril. (2026) 125:574–82. doi: 10.1016/j.fertnstert.2026.01.010 41577072

[19] Gullo G Satullo M Billone V De Paola L Petousis S Kotlik Y . The role of the genital tract microbiome in human fertility: a literature review. J Clin Med. (2025) 14:2923. doi: 10.3390/jcm14092923 40363959 PMC12072807

[20] Alemu BK Wang CC Li L Zhu Z Li Q Wang Y. Effect of preconception antibiotics exposure on female reproductive health and pregnancy outcomes: a systematic review and meta-analysis. EClinicalMedicine. (2024) 78:102935. doi: 10.1016/j.eclinm.2024.102935 39687430 PMC11647117

[21] Whittemore JC Price JM Moyers T Suchodolski JS. Effects of synbiotics on the fecal microbiome and metabolomic profiles of halthy research dogs administered antibiotics: a randomized, controlled trial. Front Vet Sci. (2021) 8:665713. doi: 10.3389/fvets.2021.665713 34124225 PMC8187564

[22] Espinosa-Gongora C Jessen LR Kieler IN Damborg P Bjørnvad CR Gudeta DD . Impact of oral amoxicillin and amoxicillin/clavulanic acid treatment on bacterial diversity and β-lactam resistance in the canine faecal microbiota. J Antimicrob Chemother. (2020) 75:351–61. doi: 10.1093/jac/dkz458 31778166

[23] Bertero A Banchi P Del Carro A Corrò M Colitti B Van Soom A . Meconium microbiota in naturally delivered canine puppies. BMC Vet Res. (2024) 20:363. doi: 10.1186/s12917-024-04225-2 39135043 PMC11318152

[24] Milani C Corrò M Drigo M Rota A. Antimicrobial resistance in bacteria from breeding dogs housed in kennels with differing neonatal mortality and use of antibiotics. Theriogenology. (2012) 78:1321–8. doi: 10.1016/j.theriogenology.2012.05.033 22898018

[25] Bertero A Corrò M Del Carro A Spagnolo E Milani C Diana A . Antimicrobial pressure in healthy breeding dogs vs household animals assessed through the resistance profile of Escherichia coli and coagulase positive Staphylococci. Vet J. (2025) 311:106337. doi: 10.1016/j.tvjl.2025.106337 40120715

[26] Horodyska I Kasperska P Michalski K Bubak J Herman I Miszczak M. Natural microbiota of dogs and cats as a source and vector of resistance genes—clinical significance. Int J Mol Sci. (2025) 26:7717. doi: 10.3390/ijms26167717 40869035 PMC12386641

[27] Song SJ Lauber C Costello EK Lozupone CA Humphrey G Berg-Lyons D . Cohabiting family members share microbiota with one another and with their dogs. Elife. (2013) 2:e00458. doi: 10.7554/eLife.00458 23599893 PMC3628085

[28] Wetzels SU Strachan CR Conrady B Wagner M Burgener IA Virányi Z . Wolves, dogs and humans in regular contact can mutually impact each other's skin microbiota. Sci Rep. (2021) 11:17106. doi: 10.1038/s41598-021-96160-7 34429455 PMC8385068

[29] Suhadolc Scholten S Slavec B Klinc P Tozon N Papic B Koprivec S. Association of mycoplasma canis with fertility disorders in dogs: a case study Supported by clinical examination, PCR, 16S microbiota profiling, and serology. Pathogens. (2024) 13:391. doi: 10.3390/pathogens13050391 38787243 PMC11123722

[30] Jagódka D Kaczorek-Łukowska E Socha PA. The prevalence of Mycoplasma canis in the vaginas of breeding bitches. J Vet Res. (2024) 68:347–53. doi: 10.2478/jvetres-2024-0054 39318523 PMC11418374

[31] Tamiozzo PJ. Mycoplasma maculosum and mycoplasma spumans associated with fertility disorders in dogs from a Bernese Mountain dog kennel. Rev Argent Microbiol. (2022) 54:39–42. doi: 10.1016/j.ram.2021.04.001 34059367

[32] Maksimović Z Maksimović A Halilbašić A Rifatbegović M. Genital mycoplasmas of healthy bitches. J Vet Diagn Invest. (2018) 30:651–3. doi: 10.1177/1040638718778745 29790451 PMC6505912

[33] Domrazek K Konieczny P Majka M Czopowicz M Jurka P. The impact of microorganisms on canine semen quality. Animals (Basel). (2024) 14:1267. doi: 10.3390/ani14091267 38731270 PMC11083039

[34] België. Koninklijk Besluit van 21 juli 2016 betreffende de voorwaarden voor het gebruik van geneesmiddelen door de dierenartsen en door de verantwoordelijken van de dieren. Belgisch Staatsblad (2016).

[35] AMCRA. Gewijzigd koninklijk besluit van 21 juli 2016 (2024). Available online at: https://www.amcra.be/nl/nieuws/gewijzigd-koninklijk-besluit-van-21-juli-2016/?lid=14306 (Accessed May 6, 2026).

[36] Nederland. Regeling diergeneesmiddelen. Staatscourant 2022, nr. 17352 (2022).

[37] Hopman NEM Hulscher M Graveland H Speksnijder DC Wagenaar JA Broens EM. Factors influencing antimicrobial prescribing by Dutch companion animal veterinarians: a qualitative study. Prev Vet Med. (2018) 158:106–13. doi: 10.1016/j.prevetmed.2018.07.013 30220383

[38] Smith M King C Davis M Dickson A Park J Smith F . Pet owner and vet interactions: exploring the drivers of AMR. Antimicrob Resist Infect Control. (2018) 7:46. doi: 10.1186/s13756-018-0341-1 29619213 PMC5879597

[39] De Mol Z Anthierens S Dewulf S Biebaut E Ringenier M Dewulf J . Exploring the complexity of antibiotic prescribing behaviour among livestock and companion animal veterinarians in Belgium. Vet Rec. (2025) 197:e271–e80. doi: 10.1002/vetr.5776 41312857

[40] Sousa A de Rago L Pinho JO Estrela M Coelho AC Oliveira PA . Understanding how veterinarians' knowledge, attitudes, and practices influence antibiotic prescription: a systematic review of survey studies. BMC Vet Res. (2025) 21:543. doi: 10.1186/s12917-025-05001-6 40993662 PMC12462370

[41] Martínez EP Golding SE van Rosmalen J Vinueza-Burgos C Verbon A van Schaik G. Antibiotic prescription patterns and non-clinical factors influencing antibiotic use by Ecuadorian veterinarians working on cattle and poultry farms: a cross-sectional study. Prev Vet Med. (2023) 213:105858. doi: 10.1016/j.prevetmed.2023.105858 36724619

[42] Vijay D Bedi JS Dhaka P Singh R Singh J Arora AK . Knowledge, attitude, and practices (KAP) survey among veterinarians, and risk factors relating to antimicrobial use and treatment failure in dairy herds of India. Antibiotics. (2021) 10:216. doi: 10.3390/antibiotics10020216 33671483 PMC7926553

[43] Postma M Speksnijder DC Jaarsma ADC Verheij TJM Wagenaar JA Dewulf J. Opinions of veterinarians on antimicrobial use in farm animals in Flanders and the Netherlands. Vet Rec. (2016) 179:68. doi: 10.1136/vr.103618 27313178

[44] McDougall S Compton C Botha N. Factors influencing antimicrobial prescribing by veterinarians and usage by dairy farmers in New Zealand. N Z Vet J. (2017) 65:84–92. doi: 10.1080/00480169.2016.1246214 27748166

[45] Coyne LA Latham SM Dawson S Donald IJ Pearson RB Smith RF . Antimicrobial use practices, attitudes and responsibilities in UK farm animal veterinary surgeons. Prev Vet Med. (2018) 161:115–26. doi: 10.1016/j.prevetmed.2018.10.021 30466652

[46] Speksnijder DC Jaarsma DA Verheij TJ Wagenaar JA. Attitudes and perceptions of Dutch veterinarians on their role in the reduction of antimicrobial use in farm animals. Prev Vet Med. (2015) 121:365–73. doi: 10.1016/j.prevetmed.2015.08.014 26341466

[47] Visschers VH Backhans A Collineau L Loesken S Nielsen EO Postma M . A comparison of pig farmers' and veterinarians' perceptions and intentions to reduce antimicrobial usage in six European Countries. Zoonoses Public Health. (2016) 63:534–44. doi: 10.1111/zph.12260 26890125

[48] Scherpenzeel CGM Santman-Berends I Lam T. Veterinarians' attitudes toward antimicrobial use and selective dry cow treatment in the Netherlands. J Dairy Sci. (2018) 101:6336–45. doi: 10.3168/jds.2017-13591 29605325

[49] Taylor DD Martin JN Morley PS Belk KE White AE Scallan Walter EJ. Survey of production animal veterinarians' prescription practices, factors influencing antimicrobial drug use, and perceptions of and attitudes toward antimicrobial resistance. J Am Vet Med Assoc. (2020) 257:87–96. doi: 10.2460/javma.257.1.87 32538697

[50] Llanos-Soto SG Vezeau N Wemette M Bulut E Greiner Safi A Moroni P . Survey of perceptions and attitudes of an international group of veterinarians regarding antibiotic use and resistance on dairy cattle farms. Prev Vet Med. (2021) 188:105253. doi: 10.1016/j.prevetmed.2020.105253 33524793 PMC10957290

[51] L'Abee-Lund TM Heiene R Friis NF Ahrens P Sørum H. Mycoplasma canis and urogenital disease in dogs in Norway. Vet Rec. (2003) 153:231–5. doi: 10.1136/vr.153.8.231 12967146

[52] Jang SS Ling GV Yamamoto R Wolf AM. Mycoplasma as a cause of canine urinary tract infection. J Am Vet Med Assoc. (1984) 185:45–7. 6746370

[53] Laber G Holzmann A. Experimentally induced mycoplasmal infection in the genital tract of the male dog: II. Andrological and microbiological investigations after exposure to mycoplasms. Theriogenology. (1977) 7:177–88. doi: 10.1016/0093-691X(77)90214-X

[54] Holzmann A Laber G Walzl H. Experimentally induced mycoplasmal infection in the genital tract of the female dog. Theriogenology. (1979) 12:355–70. doi: 10.1016/0093-691X(79)90041-4

[55] King C Smith M Currie K Dickson A Smith F Davis M . Exploring the behavioural drivers of veterinary surgeon antibiotic prescribing: a qualitative study of companion animal veterinary surgeons in the UK. BMC Vet Res. (2018) 14:332. doi: 10.1186/s12917-018-1646-2 30404649 PMC6223057

[56] Scarborough RO Sri AE Browning GF Hardefeldt LY Bailey KE. ‘Brave Enough': A qualitative study of veterinary Decisions to withhold or delay antimicrobial treatment in pets. Antibiotics (Basel). (2023) 12:540. doi: 10.3390/antibiotics12030540 36978407 PMC10044613

[57] Jin M Osman M Green BA Yang Y Ahuja A Lu Z . Evidence for the transmission of antimicrobial resistant bacteria between humans and companion animals: a scoping review. One Health. (2023) 17:100593. doi: 10.1016/j.onehlt.2023.100593 37448771 PMC10336692

[58] Joosten P Van Cleven A Sarrazin S Paepe D De Sutter A Dewulf J. Dogs and their owners have frequent and intensive contact. Int J Environ Res Public Health. (2020) 17. doi: 10.3390/ijerph17124300 32560155 PMC7345801

