# Supplementary material for: Practices and perceptions of antibiotic use in canine breeding: a survey among breeders and veterinarians
Source: Front Vet Sci. 2026 Jul 17;13:1879574. doi: 10.3389/fvets.2026.1879574 (PMC13423706; doi:10.3389/fvets.2026.1879574)
Supplement: Supplementary file 1 [file Data_Sheet_1.pdf]

Appendix 1: Antibiotic Use in Dog Breeding: Questionnaire for Breeders

General Information

Question: Do you live in Belgium or the Netherlands?
- Belgium
- The Netherlands

Question: How long have you been breeding dogs?
- Less than 5 years
- 5–10 years
- 10–20 years
- More than 20 years

Question: Which breed(s) do you currently breed or have bred in the past?
- Open answer

Question: How many litters do you breed per year?
- Maximum of 1 litter per year
- 1–3 litters per year
- 3–5 litters per year
- More than 5 litters per year

Question: Do you prefer natural mating or artificial insemination for your bitches?
- Natural mating
- Artificial insemination
- No preference

Question: What is the reason for your preference? (multiple answers possible)
- Better control and timing with artificial insemination
- Natural mating not possible (bitch does not allow it, male does not mate)
- Concern about bacterial transmission during natural mating
- Letting nature take its course
- Higher pregnancy success with natural mating
- Higher pregnancy success with artificial insemination
- Other: (open answer)

Questions on Antibiotic Use

Question: Have you ever sought advice regarding the use of antibiotics around mating/whelping?
- Yes
- No

Question: From whom did you receive this advice? (multiple answers possible)
(Displayed if "Yes" is selected)
- Veterinarian
- Other breeders
- Artificial intelligence
- Online forums

- Scientific literature
- Other online platform, namely: (open answer)
- Other: (open answer)

Question: Have you ever administered antibiotics to your bitch in the context of breeding?
- Yes
- No

Question: When did you use antibiotics? (multiple answers possible)
(Displayed if "Yes" is selected)
- Before mating
- After mating
- During pregnancy
- Around whelping
- During lactation
- Other: (open answer)

Question: What was the reason for antibiotic use? (multiple answers possible)
(Displayed if "Yes" is selected)
- To "clean" the reproductive tract before mating or whelping
- Prevention of infections
- Treatment of a confirmed infection
- Infertility/subfertility
- Advice from a veterinarian
- Recommendation from other breeders
- Other: (open answer)

Question: What was the duration of the antibiotic treatment? (multiple answers possible)
(Displayed if "Yes" is selected)
- Less than 7 days
- 7–14 days
- 15–21 days
- More than 21 days
- Additional explanation/clarification: (open answer)

Question: Which antibiotics did you use?
(Displayed if "Yes" is selected)
- Amoxicillin
- Amoxicillin-clavulanic acid
- Clindamycin
- Doxycycline
- Cephalexin
- Unknown
- Other: (open answer)

Question: Where did you obtain the antibiotics? (multiple answers possible)
(Displayed if "Yes" is selected)
- From your regular veterinarian

- From a veterinarian specialized in reproduction
- From other breeders
- Pharmacy
- Online pharmacy with prescription
- Online without prescription
- Other: (open answer)

Question: Did you follow the same antibiotic protocol for multiple bitches?
(Displayed if "Yes" is selected)
- Yes
- No

Question: Did you notice differences between litters with and without antibiotic use?
(Displayed if "Yes" is selected above)
- Yes, namely: (open answer)
- No

Question: What are your experiences/opinion regarding the outcome of antibiotic treatment? (multiple answers possible)
(Displayed if "Yes" is selected)
- Resolution of the diagnosed infection
- Leads to larger litters
- Leads to healthier puppies
- Leads to lower puppy mortality
- Leads to more pregnant bitches
- No observable effect
- Other/comments: (open answer)

Questions on Bacteriological Testing and Mycoplasma

Question: Have you ever had a bacteriological examination performed using a vaginal swab in your bitches?
- Yes
- No

Question: What was the reason for performing this test? (multiple answers possible)
(Displayed if "Yes" is selected)
- Routine screening
- History of infections
- Advice from a veterinarian
- Advice from other breeders
- Required by the owner of the male
- Other: (open answer)

Question: Have you ever had bacteriological testing performed on semen or the prepuce of your male dog?
- Yes
- No
- I do not own a male dog

Question: What was the reason for performing this test? (multiple answers possible)
(Displayed if "Yes" is selected)
- Routine screening
- History of infections
- Advice from a veterinarian
- Advice from other breeders
- Required by the owner of the bitch
- Other: (open answer)

Question: Are you familiar with Mycoplasma infections in dogs?
- Yes
- No

Question: Have you ever had your bitch tested for Mycoplasma (PCR test)?
(Displayed if "Yes" above)
- Yes
- No

Question: If Mycoplasma is detected in the vagina of the bitch, do you consider this a cause of reduced fertility?
(Displayed if "Yes" above)
- Yes
- No
- I do not know

Question: Have you ever had your male dog tested for Mycoplasma (PCR test)?
(Displayed if applicable)
- Yes
- No

Question: If your male dog tests positive for Mycoplasma, do you consider this a cause of reduced fertility?
- Yes
- No
- I do not know

Question: If Mycoplasma is detected in the vagina of the bitch, in your opinion:
- The bitch can still be used for breeding without additional measures
- The bitch can still be used for breeding, provided antibiotic treatment is given
- The bitch should be excluded from breeding
- I do not know

Question: If your male dog tests positive for Mycoplasma:
- The male can still be used for breeding without additional measures
- The male can still be used for breeding, provided antibiotic treatment is given
- The male should be excluded from breeding
- I do not know

Question: Do you have any additional comments?
- Open answer
