# Supplementary material for: Practices and perceptions of antibiotic use in canine breeding: a survey among breeders and veterinarians
Source: Front Vet Sci. 2026 Jul 17;13:1879574. doi: 10.3389/fvets.2026.1879574 (PMC13423706; doi:10.3389/fvets.2026.1879574)
Supplement: Supplementary file 2 [file Data_Sheet_2.pdf]

Appendix 2: Antibiotic Use in Dog Breeding: Questionnaire for Veterinarians

General Information

Question: How long have you been practicing as a veterinarian?
- 0–5 years
- 6–10 years
- 11–20 years
- More than 20 years

Question: Do you work in Belgium or the Netherlands?
- Belgium
- The Netherlands

Question: Which animal species do you primarily work with? (multiple answers possible)
- Companion animals
- Cattle
- Horses
- Exotic animals

Question: What is your professional setting? (multiple answers possible)
- Primary care veterinarian
- Specialist / special interest in reproduction
- Specialist / special interest in another discipline
- Solo practice
- Group practice

Question: How often do you assist bitches for mating/breeding in your practice?
- Never
- Less than 5 times per year
- 5–20 times per year
- More than 20 times per year

Questions on Antibiotic Prescription in Breeding

Question: Have you ever prescribed antibiotics to a healthy breeding bitch around the time of mating or parturition?
- Yes
- No

Question: At which stage did you prescribe antibiotics? (multiple answers possible)
(Displayed if "Yes" is selected)
- Before mating
- After mating
- During pregnancy
- Around parturition

- During lactation (excluding mastitis/metritis)
- Other: (open answer)

Question: Which antibiotics did you prescribe? (multiple answers possible)
(Displayed if "Yes" is selected)

- Amoxicillin
- Amoxicillin-clavulanic acid
- Clindamycin
- Doxycycline
- Cephalexin
- Other/additional explanation: (open answer)

Question: How often have you prescribed antibiotics to prevent infections in breeding bitches?

- Never
- Rarely (<10% of cases)
- Sometimes (10–25% of cases)
- Regularly (25–50% of cases)
- Often (>50% of cases)
- Always

Question: Which antibiotics did you prescribe for prevention? (multiple answers possible)
(Displayed if "Never" is not selected)

- Amoxicillin
- Amoxicillin-clavulanic acid
- Clindamycin
- Doxycycline
- Cephalexin
- Other/additional explanation: (open answer)

Question: How do you typically diagnose a vaginal infection? (multiple answers possible)

- Based on history (anamnesis)
- Clinical examination
- Vaginal cytology
- Vaginal bacteriology
- Other: (open answer)

Question: How often have you prescribed antibiotics to treat a confirmed vaginal infection?

- Never
- Rarely (<10%)
- Sometimes (10–25%)
- Regularly (25–50%)
- Often (>50%)
- Always

Question: Which antibiotics did you prescribe for treatment? (multiple answers possible)
(Displayed if "Never" is not selected)

- Amoxicillin
- Amoxicillin–clavulanic acid
- Clindamycin
- Doxycycline
- Cephalexin
- Other/additional explanation: (open answer)

Question: How often have you prescribed antibiotics for infertility/subfertility?

- Never
- Rarely (<10%)
- Sometimes (10–25%)
- Regularly (25–50%)
- Often (>50%)
- Always

Question: Which antibiotics did you prescribe for infertility/subfertility? (multiple answers possible)
(Displayed if "Never" is not selected)

- Amoxicillin
- Amoxicillin-clavulanic acid
- Clindamycin
- Doxycycline
- Cephalexin
- Other/additional explanation: (open answer)

Question: How often have you prescribed antibiotics following a breeder's request?

- I have never received such a request
- Never
- Rarely (<10%)
- Sometimes (10–25%)
- Regularly (25–50%)
- Often (>50%)
- Always

Question: Which antibiotics did you prescribe following breeder requests? (multiple answers possible)
(Displayed if applicable)

- Amoxicillin
- Amoxicillin-clavulanic acid
- Clindamycin
- Doxycycline
- Cephalexin
- Other/additional explanation: (open answer)

Question: Have you prescribed antibiotics to breeding bitches in other situations?
- Yes, namely: (open answer)
- No

Vaginal Bacteriology and Vaginitis

Question: How often do owners/breeders request vaginal bacteriology in healthy breeding bitches?
- Never
- Rarely (<10%)
- Sometimes (10–25%)
- Regularly (25–50%)
- Often (>50%)
- Always

Question: How often do you see bitches with signs of vaginitis?
- Never
- Rarely (<5 times per year)
- Regularly (5–20 times per year)
- Often (>20 times per year)

Question: How often do you use vaginal bacteriology to diagnose vaginitis in bitches with discharge or clinical signs (licking, irritation, pruritus)? (excluding puppy vaginitis)
- Never
- Rarely (<10%)
- Sometimes (10–25%)
- Regularly (25–50%)
- Often (>50%)
- Always

Question: How often do you use antibiotics to treat vaginitis/vaginal discharge in bitches?
- Never
- Rarely (<10%)
- Sometimes (10–25%)
- Regularly (25–50%)
- Often (>50%)
- Always

Question: Which antibiotics do you use for vaginitis/vaginal discharge? (multiple answers possible)
(Displayed if "Never" is not selected)
- Amoxicillin
- Amoxicillin–clavulanic acid
- Clindamycin
- Doxycycline
- Cephalexin
- Other/additional explanation: (open answer)

Subfertility

Question: How often do you see dogs with fertility problems in your practice?

- Never
- <5 times per year
- 5–20 times per year
- <20 times per year

Question: How often do you use genital bacteriology (vaginal/preputial/semen) in dogs with fertility problems?

- Never
- Rarely (<10%)
- Sometimes (10–25%)
- Regularly (25–50%)
- Often (>50%)
- Always

Question: How often do you prescribe antibiotics for bitches with subfertility/infertility?

- Never
- Rarely (<10%)
- Sometimes (10–25%)
- Regularly (25–50%)
- Often (>50%)
- Always

Question: Which antibiotics do you prescribe in these cases? (multiple answers possible)

- Amoxicillin
- Amoxicillin–clavulanic acid
- Clindamycin
- Doxycycline
- Cephalexin
- Other/additional explanation: (open answer)

Breeder Requests for Antibiotics

Question: How often do breeders ask for advice regarding antibiotic use around mating or parturition?

- Never
- Rarely (<10% of breeders)
- Sometimes (10–25%)
- Regularly (25–50%)
- Often (>50%)
- Always

Question: How often do breeders request antibiotics for bitches without a clear bacteriological indication (i.e., prophylactically)?

- Never
- Rarely (<10%)
- Sometimes (10–25%)
- Regularly (25–50%)
- Often (>50%)
- Always

Mycoplasma

Question: If Mycoplasma is detected in the vagina of a bitch, do you consider it a cause of (sub)infertility?

- Yes
- No
- I do not know
- Other: (open answer)

Question: If Mycoplasma is detected in the vagina of a bitch:

- The bitch can still be used for breeding without additional measures
- The bitch can be used for breeding with appropriate antibiotic treatment
- The bitch should be excluded from breeding
- I do not know
- Other: (open answer)

Question: If you detect Mycoplasma in the vagina of a bitch:

- You prescribe antibiotics
- You do not prescribe antibiotics
- Other: (open answer)

Question: If you prescribe antibiotics following Mycoplasma detection in a bitch, which product and for how long?

- Open answer

Question: If a stud dog tests positive for Mycoplasma:

- The dog can still be used for breeding without additional measures
- The dog can be used with antibiotic treatment
- The dog can be used with artificial insemination
- The dog should be excluded from breeding
- I do not know
- Other: (open answer)

Question: If you detect Mycoplasma in a stud dog:

- You prescribe antibiotics
- You do not prescribe antibiotics
- Other: (open answer)

Question: If you prescribe antibiotics following Mycoplasma detection in a stud dog, which product and for how long?

- Open answer

Question: How often do you receive questions about Mycoplasma in relation to fertility or vaginal disorders?

- Never
- Rarely (<10%)
- Sometimes (10–25%)
- Regularly (25–50%)
- Often (>50%)
- Always

Question: How often do you perform Mycoplasma PCR diagnostics on vaginal swabs from breeding bitches?

- Never
- Rarely (<10%)
- Sometimes (10–25%)
- Regularly (25–50%)
- Often (>50%)
- Always

Question: How often do you perform Mycoplasma PCR diagnostics on swabs from stud dogs?

- Never
- Rarely (<10%)
- Sometimes (10–25%)
- Regularly (25–50%)
- Often (>50%)
- Always

Question: Do you have any additional comments?

- Open answer
